# Seasonal Hydrological Loading in Southern Tibet Detected by Joint Analysis of GPS and GRACE

**DOI:** 10.3390/s151229815

**Published:** 2015-12-04

**Authors:** Rong Zou, Qi Wang, Jeffrey T. Freymueller, Markku Poutanen, Xuelian Cao, Caihong Zhang, Shaomin Yang, Ping He

**Affiliations:** 1 Hubei Subsurface Multi-Scale Imaging Key Laboratory, Institute of Geophysics & Geomatics, China University of Geosciences (Wuhan), Wuhan 430074, China; zourong@cug.edu.cn (R.Z.); caoxuelian@cug.edu.cn (X.C.); phe@cug.edu.cn (P.H.); 2 Finnish Geospatial Research Institute FGI, Geodeetinrinne 2, Masala 02430, Finland; markku.poutanen@nls.fi; 3 Geophysical Institute, University of Alaska Fairbanks, Fairbanks, AK 99775, USA; jeff.freymueller@gi.alaska.edu; 4 Institute of seismology, China Earthquake Administration & Hubei Earthquake Administration, Wuhan 430071, China; stellazch@gmail.com (C.Z.); whgpsyang@gmail.com (S.Y.)

**Keywords:** seasonal variations, GRACE, GPS, vertical deformation, horizontal deformation

## Abstract

In southern Tibet, ongoing vertical and horizontal motions due to the collision between India and Eurasia are monitored by large numbers of global positioning system (GPS) continuous and campaign sites installed in the past decade. Displacements measured by GPS usually include tectonic deformation as well as non-tectonic, time-dependent signals. To estimate the regional long-term tectonic deformation using GPS more precisely, seasonal elastic deformation signals associated with surface loading must be removed from the observations. In this study, we focus on seasonal variation in vertical and horizontal motions of southern Tibet by performing a joint analysis of GRACE (Gravity Recovery and Climate Experiment) and GPS data, not only using continuous sites but also GPS campaign-mode sites. We found that the GPS-observed and GRACE-modeled seasonal oscillations are in good agreements, and a seasonal displacement model demonstrates that the main reason for seasonal variations in southern Tibet is from the summer monsoon and its precipitation. The biggest loading appears from July to August in the summer season. Vertical deformations observed by GPS and modeled by GRACE are two to three times larger than horizontal oscillations, and the north components demonstrate larger amplitudes than the east components. We corrected the GPS position time series using the GRACE-modeled seasonal variations, which gives significant reductions in the misfit and weighted root-mean-squares (WRMS). Misfit ($\chi^{2}$ divided by degree of freedom) reductions for campaign sites range between 20% and 56% for the vertical component, and are much smaller for the horizontal components. Moreover, time series of continuous GPS (cGPS) sites near the 2015 Nepal earthquakes must be corrected using appropriate models of seasonal loading for analyzing postseismic deformation to avoid biasing estimates of the postseismic relaxation.

## 1. Introduction

**Figure 1:**
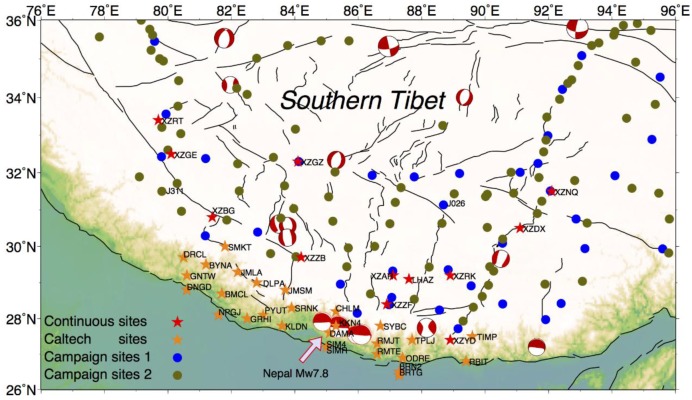
The distribution of GPS sites used in this paper. The stars are continuous GPS sites, including Crustal Movement Observation Network of China, CMONOC sites (CMONOC I and II) in red and Caltech sites in orange. The dots correspond to campaign sites with the blue for CMONOC I and the dark green for CMONOC II. The beach balls delineate recent major earthquakes in southern Tibet, including the Mw7.8 Nepal earthquake and major aftershocks in 2015, from the Global Centroid-Moment-Tensor (CMT) catalog.

The ongoing crustal deformation of the Tibetan Plateau results from the collision between India and Eurasia. The Indian plate has been moving northward at a rate of ~4 cm/year with respect to Siberia. About half of the continental convergence is accommodated by crustal shortening across the southern edge of the Tibetan Plateau [1,2,3,4]. As a result, the Himalaya, the highest mountain ranges on Earth, has been the site of large earthquakes, including several M ~8 devastating earthquakes in the past centuries [5]. For example, a massive Mw 7.8 earthquake struck the Nepal Himalaya in April 2015 [6,7,8], causing about 7500 deaths and injuries to more than 14,000 people injured (Figure 1). It is very important to understand active deformation in this region.

It is well known that the present-day deformation manifest geodetically in the Himalaya and southern Tibet contains not only signals associated with tectonic processes such as earthquake deformation but also transient processes caused by surface hydrology [9]. Seasonal oscillation in vertical motion associated with the summer monsoon is estimated to be up to several centimeters peak-to-peak [10]. The seasonal variation must be understood thoroughly in order to isolate tectonic signals from geodetic time series for preseismic or postseismic deformation. The GRACE mission, designed for monitoring time-dependent gravity changes from space, provides a unique opportunity to estimate seasonal deformation in terms of surface water loading [11], which is retrieved from the global gravity field sampled monthly at a spatial scale larger than 330 km.

The elastic deformation due to surface water transport is detected in both GPS [12,13,14,15,16,17,18,19,20,21] and GRACE time series [22,23]. The loading signal is especially pronounced in the vertical component of site position, and the change in water thickness as a function of location in the westernmost U.S. has been estimated from GPS vertical displacements [24,25], advancing the application of GPS to hydrology. GPS-observed seasonal variations in site coordinates have been compared with GRACE-derived loading deformation on global [26,27,28] and regional scales for Central America [29], Europe [30], Fennoscandia [31,32], Greenland [33], the Nepal Himalaya [10], West Africa [34], southern Alaska [35], and the North China Plain [36]. Consistency at seasonal periods between GPS and GRACE measurements has been observed generally in regions where surface water transport is significant over larger spatial scales. Long-term trends are usually not in agreement because of local effects such as tectonic deformation, and because the Glacial Isostatic Adjustment (GIA) signal in gravity and displacement is not identical except for purely elastic deformation.

Earlier studies focused on vertical components of cGPS sites in consideration of the larger signal in the vertical and the need for continuous observations to compare independent measures of seasonal variation. Recently, horizontal components of cGPS time series also have been examined to constrain surface loading changes in combination with the vertical components [37,38,39]. GPS sites in southern Tibet are surveyed mainly in campaign mode for monitoring crustal deformation. The position time series are affected non-uniformly by surface loading because observations at any site, which were made every 2–3 years, were not conducted at the same time of year each survey. Traditionally, the estimation of tectonic motion rates, for example for the horizontal velocity field for southern Tibet [40,41,42], is made assuming that seasonal effects can be ignored. However, accurate determination of the small ongoing uplift of the Tibetan Plateau requires removing seasonal variations in vertical components from site position time series [9,43]. Seasonal oscillations in GPS site time series also can bias estimates of postseismic deformation, especially in the critical first months after an earthquake. We investigate tectonic and hydrologic deformation at GPS sites in southern Tibet, focusing on removing seasonal signals in GPS time series for a robust determination of tectonic deformation. This study represents a considerable complement to the previous works that were based exclusively on analyzing the Nepal continuous GPS network because new sites in southern Tibet, China are considered, and numerous time series of campaign sites are analyzed. In particular, we analyze how removing seasonal hydrologic signals from GPS site time series impacts estimates of the postseismic transient following the 2015 Mw 7.8 Gorkha earthquake [6].

## 2. Geodetic Measurements

### 2.1. Continuous and Campaign GPS Observations and Data Processing

Former and current cGPS and campaign stations in southern Tibet (Figure 1) are analyzed in this study. The CMONOC I included one cGPS site and 37 campaign-mode GPS sites here, while there were 13 cGPS sites and 87 campaign-mode GPS sites from the Tectonic and Environment Observation Network of Mainland China (also called CMONOC II). Additional sites have been installed and measured by a variety of other organizations. For example, we used 28 continuous GPS sites in Nepal which were installed by Caltech and other groups these data are available from UNAVCO archive and Caltech’s Tectonics Observatory website. Half of the 13 continuous GPS sites of CMONOC II started to record measurements in August 2011, and the observations of the campaign-mode sites belonging to CMONOC II were surveyed in 2009, 2011 and 2013. The details of the data are in Table 1. Because of the hard-to-access mountainous terrain in southern Tibet, there are not many continuous GPS sites and most of the sites are observed in campaign mode.

**Table 1:** GPS data in southern Tibet used in this study.

Stations	Number	Time Span	Organization
Continuous stations	1	1998–2014	CMONOC I
	13	2011–2015	CMONOC II
	28	1999–2015	Caltech
Campaign stations	36	1999–2013	CMONOC I
	87	2009–2013	CMONOC II

We used the GIPSY/OASIS software (version goa-5.0) to process the GPS data and estimate station coordinates for all sites in precise point positioning (PPP) mode. The data analysis follows the procedures described in Fu and Freymueller [10]. We used JPL’s reanalysis orbit and clock products, which were determined using a consistent set of models over the entire time span, including absolute antenna phase center models (igs08.atx) for both GPS receiver and satellite antennas [44] that were used in generating the orbit and clock products. The “non-fiducial” version of JPL’s orbit and clock products was adopted, which are the product of a free network solution in which no coordinates were fixed.

We estimated PPP solutions for all sites shown in Figure 1 on each day along with a global set of sites, and then combined the stations to form a solution for the day. Each daily solution is aligned to ITRF2008, as JPL’s “non-fiducial” solution is in an indeterminate frame due to the loose constraints applied in their master orbit and clock solution [45,46]. We used a standard seven-parameter similarity transformation with about 160 sites distributed all over the world to align this reference frame to ITRF2008 [47]. We then separated the stations in three groups (the CMONOC I, CMONOC II and Caltech Nepal networks) to analyze each set independently. In this study, we do not correct the atmospheric and non-tidal ocean loading effects during the GPS data processing.

### 2.2. Analysis and Processing of GRACE Measurements

We employed the GRACE Level-2 Release-05 (RL05) of monthly gravity field products, GRACE Satellite only Model (GSM) solution files, provided by the University of Texas Center for Space Research (UTCSR). Those GSM files are in the form of spherical harmonics coefficients (SHCs) up to degree 60, from August 2002 to February 2015. Displacements due to the changing mass load can be computed from spherical harmonic coefficients for the surface load and the load Love numbers [30,48,49].

(1) $$\begin{array}{c} ∆n=-R\overset{\infty }{\underset{l=1}{\sum }}\overset{l}{\underset{m=0}{\sum }}{\bar{P}}_{lm}\left(cos\theta \right)\left(∆C_{lm}\cos\left(m\lambda \right)+∆S_{lm}sin\left(m\lambda \right)\right)\frac{l_{l}^{\prime }}{1+k_{l}^{\prime }}\\ ∆e=\frac{R}{sin\theta }\overset{\infty }{\underset{l=1}{\sum }}\overset{l}{\underset{m=0}{\sum }}\left(cos\theta \right)\left(-∆C_{lm}\sin\left(m\lambda \right)+∆S_{lm}cos\left(m\lambda \right)\right)\frac{l_{l}^{\prime }}{1+k_{l}^{\prime }}\;\\ ∆h=R\overset{\infty }{\underset{l=1}{\sum }}\overset{l}{\underset{m=0}{\sum }}\left(cos\theta \right)\left(∆C_{lm}\cos\left(m\lambda \right)+∆S_{lm}sin\left(m\lambda \right)\right)\frac{h_{l}^{\prime }}{1+k_{l}^{\prime }}\; \end{array}$$

where ∆n, ∆e and ∆h represent the displacement of Earth surface in the north, east and vertical directions, respectively; R is the average Earth radius; ${\bar{P}}_{lm}$ are fully normalized Legendre functions of degree l and order m; $∆C_{lm}$ and $∆S_{lm}$ represent the residual spherical harmonics coefficients of the destriped and smoothed gravity field relative to a long-term average, respectively; and $h_{l}^{\prime }$ and $k_{l}^{\prime }$ are the load Love numbers of degree l. Here we adopt the load Love numbers from Farrell [50].

We applied Gaussian smoothing for the GRACE products with an average radius of 400 km. We replaced the degree-1 gravity coefficients with results obtained by Swenson [51], which is based on ocean and atmospheric models and GRACE coefficients for degree 2 and higher. The C20 terms with results from the analysis of Satellite Laser Ranging (SLR) data to five geodetic satellites: LAGEOS-1 and 2, Starlette, Stella and Ajisai [52], whose background gravity model used in the SLR analysis is consistent with the RL05 processing. In order to maintain consistency with the loading signals contained in the GPS time series, we added the GRACE Atmosphere and Ocean De-aliasing Level-1B (AOD1B) product (GAC solution) to the CSR GSM solution to add back the effects of atmospheric loading, non-tidal ocean loading and other loading effects present in the GPS time series, primarily because those effects are not as easily removed from the GPS time series.

**Figure 2:**
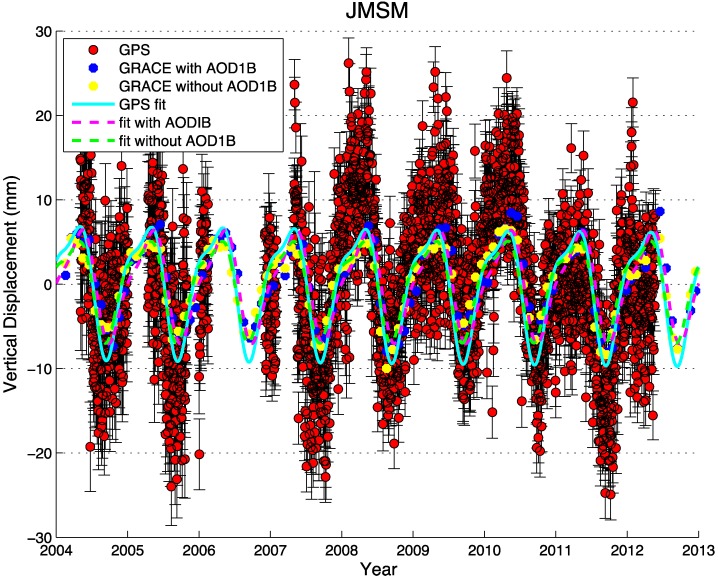
Seasonal variations of JMSM station for example to compare the GPS observations and GRACE models with and without AOD1B correction. All the time series used here are detrended.

We then computed 3D displacement time series at all GPS sites based on this load model, using the load Love numbers from Farrell [50], which are computed relative to the center of mass of the solid earth. Seasonal variations for the loading displacements were computed from these model-predicted time series. Figure 2 shows an example GPS station JMSM (Jomsom, Nepal) for the comparison of GRACE-modeled vertical displacements with and without AOD1B model added to the GRACE solutions. The GRACE models with AOD1B (the purple dashed line) agree with the GPS seasonal variations (the cyan line) a little bit better (the average correlation coefficient between GPS and GRACE increase 0.08) than the models without AOD1B (the green dashed line), the difference is small, mainly because the southern Tibet is far from the ocean. However, Fu* et al.* [35] showed that using AOD1B is important for consistency so it is necessary to consider the atmosphere and non-tidal ocean loading during the comparison of GPS observations and GRACE solutions. The earlier study by Fu* et al.* [10] in the Nepal Himalaya neglected to add AOD1B, so their GPS-GRACE comparisons were not fully consistent. We also compared the AOD1B effect in horizontal displacements. The difference is not readily apparent on the present noise level, because the amplitude of seasonal variation in the horizontal component is much less than in the vertical.

### 2.3. Comparison between Seasonal Variations in GPS and GRACE

**Figure 3:**
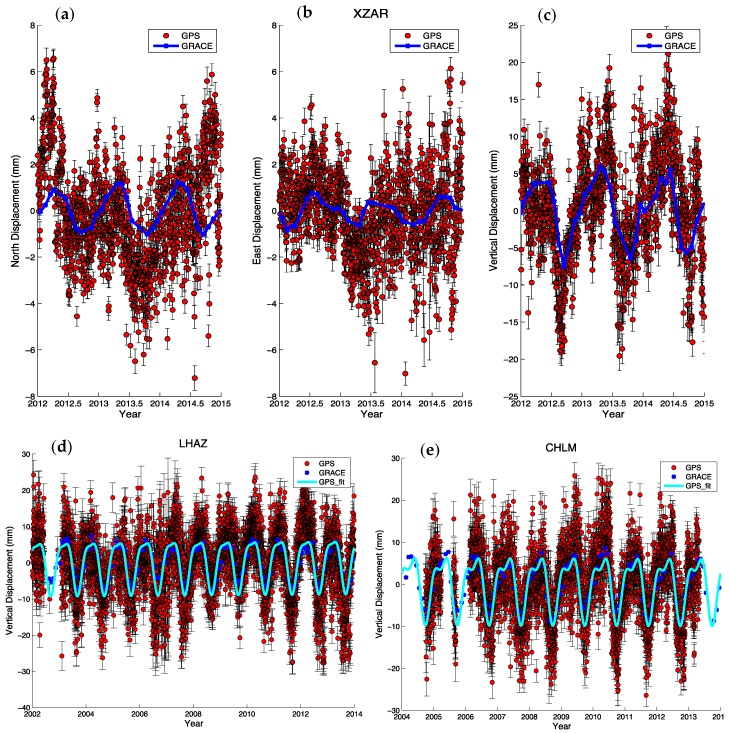
Comparison between GPS and GRACE detrended time series at three sites: XZAR in three components (**a**-**c**), LHAZ (**d**) and CHLM (**e**) in vertical components.

Figure 3 shows the comparison of detrended GPS time series and the GRACE measurements for three GPS sites. The strong correspondence between the GPS-observed and GRACE-modeled seasonal variations is clear for all sites. The site XZAR (Ang Ren in Tibet) is newly established in CMONOC II; it began to record observations in the late 2011, and the period shown here is from 2012 to the end of 2014. Large seasonal variations appear in the vertical direction. As for the horizontal directions, the seasonal variations are much larger in the north direction than in the east, but the north component variations are still about a factor of three smaller than the vertical. Another two sites shown in Figure 3 are cGPS sites with long observation histories, the site LHAZ located in Lhasa, China, and the site CHLM located in Nepal. At both these sites, the GRACE loading model matches the GPS measurements in amplitude, phase and the shape of the waveform; the scatter of the GPS observations is clearly higher. The agreement between GPS and GRACE is more clearly seen when the seasonal fit to the GPS is compared to the GRACE predictions, due to the noise reduction of the seasonal fit.

### 2.4. Modeling of Seasonal Variations

We derive seasonal loading models for each site from the time series of loading displacements predicted by the GRACE load model using a nonparametric approach [18]. When comparing amplitudes or phases of the loading signals, we also apply the same methods to the observed GPS time series. Some of the potential seasonal variation contributors do not exhibit exact sinusoidal form, and a nonparametric approach is more suitable for such situations. Using a nonparametric approach to model seasonal variation avoids making any assumptions about the contributors other than an annual period [18,53].

**Figure 4:**
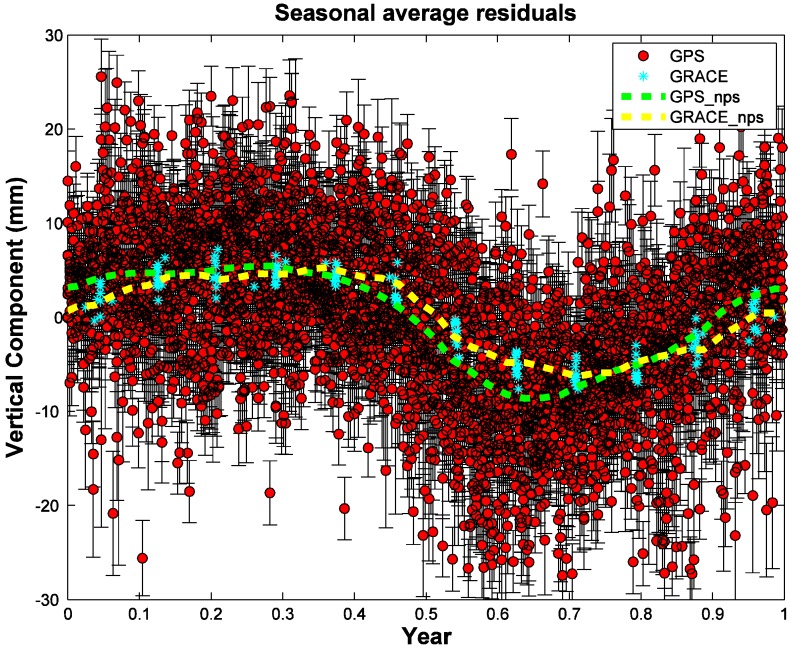
Estimated seasonal variations between GPS and GRACE detrended time series in vertical components at LHAZ, plotted by fractional year. We use the nonparametric seasonal approach (nps) to model the GRACE loading effects, the red circles are the GPS measurements, the cyan stars are the GACE predictions, and the green and yellow dashed lines show the seasonal nonparametric models for GPS and GRACE, respectively.

Figure 4 illustrates how the loading models are derived from GRACE displacement predictions using the nonparametric method. Observations from all years are averaged based on their fractional time of year and derive the average seasonal displacement [18]. The summer monsoon brings significant rainfall and increased surface mass loading, so the seasonal surface load reaches its highest value and the vertical position reaches its lowest point in July to August. The timing indicates that the rainfall loading dominates the seasonal deformation at LHAZ.

We adopt the WRMS reduction percentage to assess the correlation between GPS-observed and GRACE-modeled seasonal displacement. We remove the GRACE-modeled seasonal deformation from GPS-observed position time series, and compute the reduction percentage of WRMS based on following equation [30].

(2) $$WRMS_{Reduction}=\frac{WRMS_{GPS}-WRMS_{GPS-GRACE}}{WRMS_{GPS}}$$

where $WRMS_{GPS}$ is the WRMS of the GPS (detrended) time series, including all seasonal variations; $WRMS_{GPS-GRACE}$ is the WRMS of the GPS time series with seasonal oscillation corrected by GRACE-modeled seasonal displacements.

## 3. Results and Discussion

### 3.1. Correcting Hydrological Loading Deformation by GRACE Data

The summer monsoon brings significant rainfall that inevitably induces additional mass loading on southern Tibet and the Himalaya, and previous studies have demonstrated that rainfall in the summer is the biggest source of seasonal variations in the GPS time series. In order to remove hydrology-induced deformation signals and better measure tectonic phenomena, we used GRACE measurements to remove the hydrological signals from the time series based on the seasonal loading model mentioned above. In the following sections, we will analyze the effects of seasonal variations in the vertical and horizontal directions. We validate the approach using the continuous GPS sites, and then apply it to the campaign data.

#### 3.1.1. Vertical Deformation

**Figure 5:**
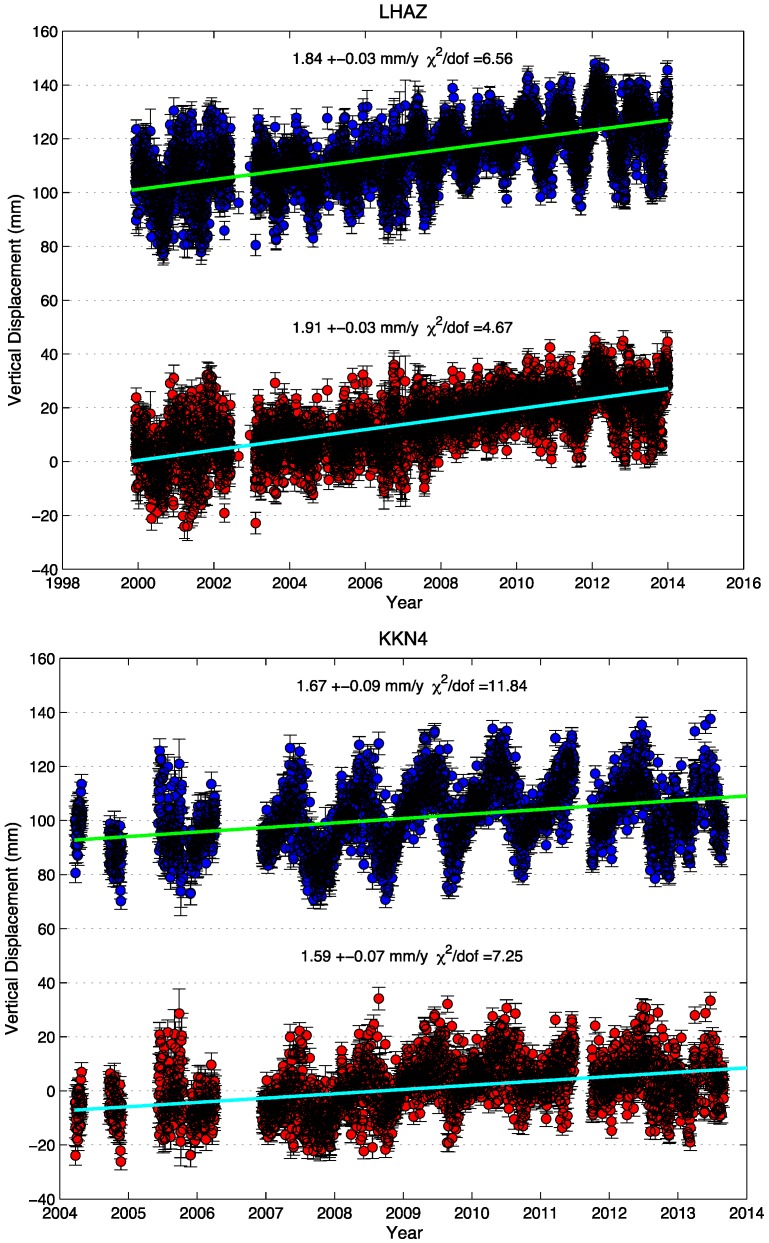
Comparison of linear fit between actual GPS observed time series (blue) and corrected time series (red) with seasonal effects removed, based on GRACE-derived seasonal variations.

GRACE-modeled seasonal variations for the vertical deformation can be used to correct seasonal effects in the GPS time series. We choose the continuous GPS sites LHAZ and KKN4 (Kahani 4, located in Kathmandu Nepal) as examples. In Figure 5, we compared the vertical displacements before and after the removal of hydrological loading effects using GRACE-modeled seasonal variations. The misfit shows the reduction in misfit from applying this correction (errors in the GPS time series are assumed to be uncorrelated in these calculations). The misfit between the data and a linear fit decreased by 30% (from 6.56 to 4.67) and 39% (from 11.84 to 7.25) in the vertical for LHAZ and KKN4, respectively. For all the cGPS sites of CMONOC II, the highest misfit reduction is 42%, and the smallest misfit reduction is 16%, the average misfit reduction is 25.9%, and for all the cGPS sites located in Nepal the corresponding value is from 11% to 44%; the average for Nepal is 28%. The seasonal hydrological mass changes are large in the Himalaya and southern Tibet, where the peak-to peak seasonal amplitude can reach more than 2 cm.

#### 3.1.2. Horizontal Deformation

Most earlier studies focused on the vertical deformation because of its large amplitude. Wahr * et al.* [37] demonstrated that the horizontal displacement is valuable in which they constrain the location of load changes better than the vertical and therefore augment the vertical observations [38]. To characterize the horizontal oscillations, analyze was also performed in both GPS-observed and GRACE-modeled horizontal seasonal variations in the north and east components, respectively. GPS and GRACE show good correlation, the average correlation coefficient is about 0.85 and 0.7; they both record coherent horizontal seasonal ground movements. The north component shows a little bit larger (0.5–2.5 mm) amplitude than east component. The good correlation between these two geodetic measurements confirms that the seasonal crustal deformation is hydrological seasonal mass loading variation.

The reduction of WRMS in the north component is about 30%, but in the east component is not so good, about two-thirds of the sites’ reduction in the east component is about 5%–10%, and for the other third, it is even negative (meaning increased WRMS after correction). This reflects a combination of the smaller horizontal signal, especially in the east component, possible systematic errors in the GPS data, and the spatial smoothing of the GRACE data.

### 3.2. Seasonal Variations in Campaign GPS Sites

Seasonal variations also exist in campaign GPS sites if the observations are not made at the same time of year, but they cannot be removed except using an external calibration model because there are too few observations to estimate seasonal corrections from the data, neglecting the seasonal impact can bias the estimated linear velocities [54]. We can use the GRACE measurements to model the seasonal deformation and correct the seasonal effects in campaign GPS measurements. The change in misfit is used to assess the quality of the correction.

**Figure 6:**
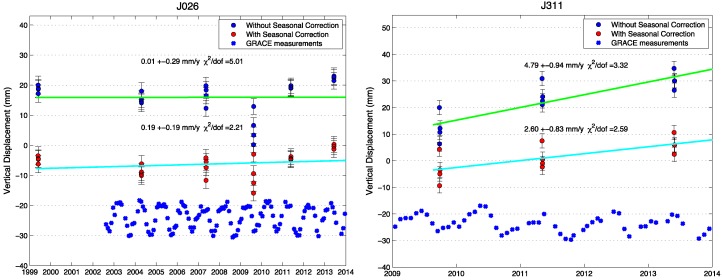
Time series of two campaign GPS sites (J311 and J026, their locations are illustrated in Figure 1). The original GPS time series (top, dark blue), corrected time series (middle, red) with seasonal loading deformation removed by the GRACE model, and the GRACE measurements demonstrate the apparent seasonal oscillations (bottom, light blue).

The good results for cGPS sites indicate that the same method could be used for GPS campaign measurements. We tested this hypothesis for campaign GPS sites, and Figure 6 shows two examples, J026 with a long measurement history and J311 newly established in CMONOC II. The misfit decreases by 56% (from 5.01 to 2.21) and 22% (from 3.32 to 2.59) in the vertical for J026 and J311, respectively. The misfit reduction is obvious (the highest misfit is 56%, and the smallest one is 20%) in the vertical for all campaign sites where measurements were resurveyed at different times of year. In some cases, the estimated vertical rate changes significantly (for example, J311) when the corrections are applied. This tends to occur for sites with fewer measurements and a shorter time span (in contrast, for the continuous sites the estimated rate rarely changed).

### 3.3. Impacts on Post-Earthquake Measurements for the 2015 Nepal Earthquake

**Figure 7:**
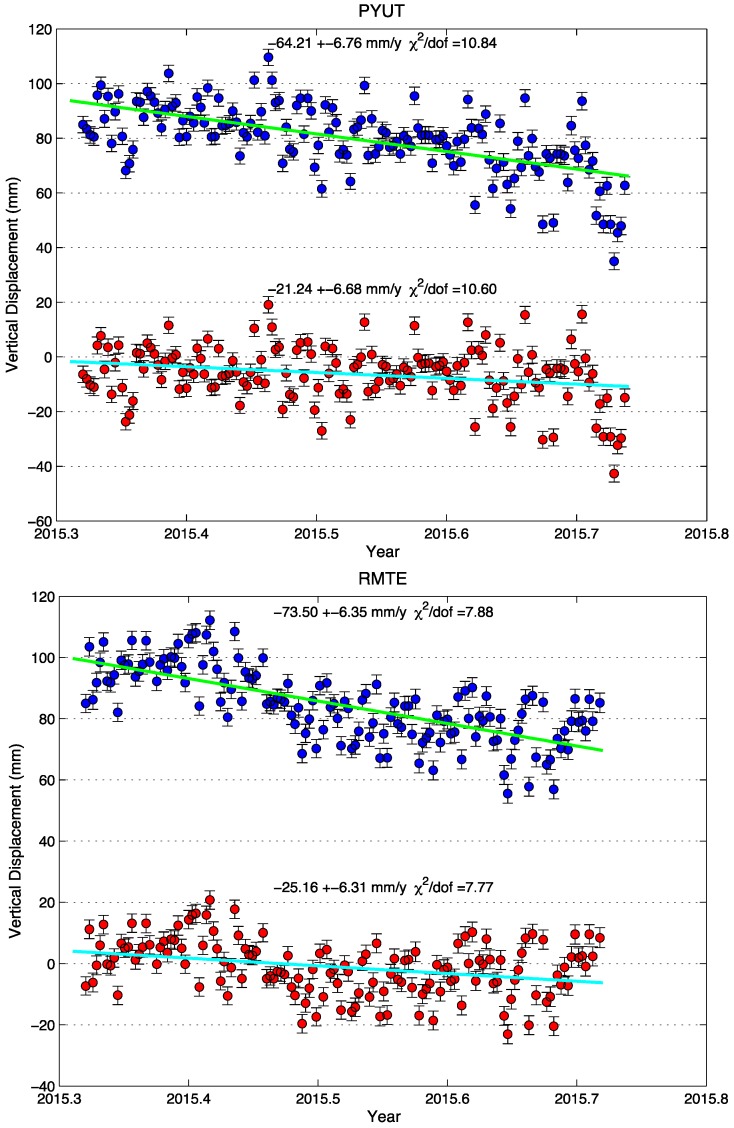
Comparison of linear fit between original GPS observed postseismic (blue) and corrected postseismic (red) with seasonal effects removed, based on GRACE-derived seasonal model.

The 2015 Mw7.8 Gorkha earthquake occurred in late April [6]. In the first half-year since this thrusting earthquake, postseismic displacements for sites in the Himalaya and southern Tibet have been mainly to the south, in the same direction as the coseismic displacement. Because this is in the same direction as the largest horizontal seasonal oscillation, and because the impact of an annual period oscillation on the estimated rate is greatest when the data span is half a cycle, the seasonal displacements can have a significant impact on the early postseismic displacements. Figure 7 shows the data from two sites located in the foothills of Nepal Himalaya, where the coseismic slip did not reach and where shallow afterslip might be expected to occur. The N–S components of both time series in PYUT (Pyuthan, located in Nepal and installed on 30 March 2011) and RMTE (Ramtie, located in Nepal and installed on 23 September 2008), show obviously increased southward motions at the level of 7–8 mm/year with respect to those in the interseismic time, which might be interpreted as evidence for shallow afterslip.

Postseismic deformation is time dependent, and post-earthquake GPS displacements often display a logarithmic or exponential decay with time, or a similar time-dependent relaxation. The shape of these relaxation curves is widely used to infer the dominant postseismic mechanisms and time scales. The presence of seasonal loading deformation can distort the apparent shape of these relaxation curves, and potentially impact the conclusions drawn about postseismic processes or time scales. It is noted that the seasonal variation over a period of a half-year containing the summer amounts to 15 mm in the vertical component and 5 mm in the horizontal component. For the Nepal earthquake, the seasonal displacements will substantially change the apparent rate of postseismic deformation if they are not removed using an appropriate model, and thus bias estimates of postseismic relaxation parameters and mechanisms. Thus it is prerequisite to deduce realistic surface displacements from GPS time series to delineate its spatial pattern and estimate the amount.

Toward that end, we used the GRACE-based seasonal model to correct the original time series for a half-year duration (Figure 7). The N-S components of two corrected time series show a northward motion at rates similar as the interseismic one, suggesting a minor postseismic transient of 2–4 mm/year in this direction due to either afterslip or lithosphere relaxation. Thus we conclude that significant shallow afterslip did not follow this event. The weak signal inferred from the horizontal time series does not identify which postseismic deformation mechanism prevails.

Postseismic vertical displacements will depend on the site location and the dominant mechanism of postseismic deformation. Figure 7 shows that the two sites have been subsiding monotonically with cumulative displacements of 25–40 mm in the six months after the earthquake, highlighting a compound effect imparted by the summer rainfall loading and the postseismic deformation associated with this event. The corrected time series show the same sign but the cumulative six-month displacement is reduced at least by a factor of two in vertical component of the originals. Nevertheless, the 21–25 mm/year of postseismic subsidence at the foothills of central Nepal Himalaya are opposite to the uplift in the interseismic period and as such are actually one order of magnitude larger than the 2–4 mm/year of postseismic northward motion estimated at PYUT and RMTE, demonstrating the importance of the vertical deformation to help distinguish the dominating mechanism. Detailed analysis of the transient signals in terms of pattern, amount and nature is beyond the scope of this paper. It is however noted that the analysis cannot be achieved without a robust estimation of seasonal perturbations on the cGPS time series from rainfall loading, in particular given that the postseismic transients will be weaker in the following years.

## 4. Conclusions

GRACE-modeled displacements due to seasonal hydrological loading show high correlation with GPS observed seasonal variations, which confirms that the hydrological mass cycle is the main cause of seasonal deformation in southern Tibet. From the shape of modeled seasonal variations, we find that the biggest surface mass anomaly appears during July to August and the smallest one appears during February. This indicates that the summer monsoon and induced rainfall are the most important reason of the loading change in southern Tibet.

We use the misfit and WRMS reduction after removing the GRACE-modeled seasonal deformation from the GPS position time series to evaluate the correction of seasonal effects by GRACE. For CMONOC II sites, in vertical component, the range of misfit reduction is from 15%–38%, for CMONOC I sites (with longer time series), it is from 20%–56%. The north component has larger seasonal variations than the east component, and the correlation between GPS-observed and GRACE-modeled seasonal displacement shows that the correlation in the north component is better than in the east component.

The results presented in this paper demonstrate that loading models based on GRACE measurements can effectively remove the seasonal signals in both continuous and campaign-mode GPS observations in this region with very large seasonal hydrological loading. We also demonstrate that the seasonal variation from a GPS site in southern Tibet agrees well with the seasonal oscillations estimated from the GRACE time-variable monthly gravity field. The seasonal variations in the GPS time series amount to up to 15 mm in vertical direction that cannot be ignored when GPS series in this region are used to determine postseismic deformation associated with the 2015 Nepal earthquake. Further studies should consider the spatial smoothing impact of GRACE solutions, the effects of non-tidal ocean, snow, groundwater, permafrost freezing and thawing in the GPS position time series, the GIA effect, which can cause both gravity change and vertical motion, the long-term vertical deformation in this region [55], as well as the Common Mode Error caused by seasonal signals in geodetic time series.
